# Immunomodulatory Effects of Vitamin D in Thyroid Diseases

**DOI:** 10.3390/nu12051444

**Published:** 2020-05-16

**Authors:** Chiara Mele, Marina Caputo, Alessandro Bisceglia, Maria Teresa Samà, Marco Zavattaro, Gianluca Aimaretti, Loredana Pagano, Flavia Prodam, Paolo Marzullo

**Affiliations:** 1 Department of Translational Medicine, University of Piemonte Orientale UPO, 28100 Novara, Italy; chiara.mele1989@gmail.com (C.M.); gianluca.aimaretti@med.uniupo.it (G.A.); 2 Division of General Medicine, S. Giuseppe Hospital, I.R.C.C.S. Istituto Auxologico Italiano, 28824 Verbania, Italy; 3 Department of Health Sciences, University of Piemonte Orientale UPO, 28100 Novara, Italy; marina.caputo@uniupo.it (M.C.); flavia.prodam@med.uniupo.it (F.P.); 4 Division of Endocrinology, University Hospital “Maggiore della Carità”, 28100 Novara, Italy; mariateresa.sama@maggioreosp.novara.it (M.T.S.); marco.zavattaro@med.uniupo.it (M.Z.); 5 Division of Endocrinology, Diabetology and Metabolism, Department of Medical Sciences, University of Turin, 10126 Turin, Italy; bisce90@hotmail.it (A.B.); loredana.pagano@med.uniupo.it (L.P.)

**Keywords:** vitamin D, immune system, thyroid autoimmunity, thyroid cancer

## Abstract

Vitamin D is a secosteroid with a pleiotropic role in multiple physiological processes. Besides the well-known activity on bone homeostasis, recent studies suggested a peculiar role of vitamin D in different non-skeletal pathways, including a key role in the modulation of immune responses. Recent evidences demonstrated that vitamin D acts on innate and adaptative immunity and seems to exert an immunomodulating action on autoimmune diseases and cancers. Several studies demonstrated a relationship between vitamin D deficiency, autoimmune thyroid disorders, and thyroid cancer. This review aims to summarize the evidences on the immunomodulatory effect of vitamin D on thyroid diseases.

## 1. Introduction

Vitamin D is a secosteroidal hormone precursor. This term encompasses several compounds, but the most represented isoforms are Ergocalciferol (or vitamin D2), available in plants, and Cholecalciferol (or vitamin D3), synthesized at the skin level from 7-dehydrocholesterol after exposure to ultraviolet B (UVB) radiation [1,2]. Vitamin D binding protein transports vitamin D isoforms to the liver, where they are converted by 25-hydroxylase enzyme to 25-hydroxyvitamin D2 (25(OH)D2) and D3 (25(OH)D3), which are the main circulating isoforms of vitamin D and reflect vitamin D status [1,3]. Considering that D3 is the most represented isoform in humans [4], from now on we will conventionally use the terminology associated with this isoform.

At physiological concentrations, 25(OH)D3 is inactive, needing to be converted into the active forms 1,25-dihydroxyvitamin D3 (1,25(OH)2D3) by 1*α*-hydroxylase enzyme (encoded by CYP27B1) in the kidneys. 1*α*-hydroxylase activity is regulated by parathyroid hormone (PTH) levels, while high 1,25(OH)2D3 levels and fibroblast growth factor 23 (FGF23) exert a negative feedback. 1*α*-hydroxylase is also expressed in extra-renal sites, like bone, skin, colon, brain, and immune cells, where its regulation is independent of PTH. Inactivation of both 25(OH)D3 and 1,25(OH)2D3 is performed by 24-hydroxylase [1,2,5].

1,25(OH)2D3 binds to the Vitamin D receptor (VDR), a member of the nuclear hormone receptors family, acting on the vitamin D response element (VDRE) to control the expression of multiple genes, including those involved in the regulation of cellular cycle and angiogenesis [6]. Moreover, the existence of a membrane-bound VDR has been hypothesized mediating non-genomic, rapid effects of 1,25(OH)2D3 [7].

1,25(OH)2D3 has been recognized as a key hormone in the regulation of the musculoskeletal homeostasis. However, extra-skeletal effects of 1,25(OH)2D3 have been attracting interest in the last years, after discovering the presence of VDR in many tissue types [8,9]. Thus, different roles have been attributed to vitamin D: it contributes to the development, protection, transmission, and plasticity of the nervous system, downregulates the renin-angiotensin-aldosterone system, exerts a protective role on the vascular endothelium, and improves insulin sensitivity [10,11,12,13]. For these physiological evidences, vitamin D status has been proposed as a biomarker of general health and hypovitaminosis D has been correlated to the presence of metabolic syndrome, cardiovascular diseases, cancers, infections, neuromuscular disorders, and all-cause mortality [14,15,16].

**Figure 1:**
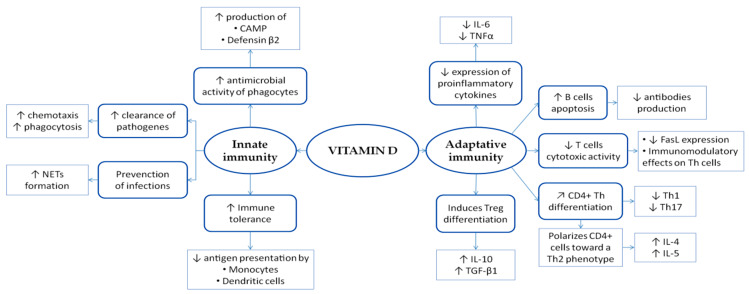
Scheme of vitamin D modulating role in immune response. As detailed in the text, vitamin D interferes with innate and adaptative immunity through different mechanisms. Arrows illustrate increase (↑), decrease (↓) or regulation/modulation (↗) of specific actions, processes, cells, or molecules. Abbreviations: CAMP, cathelicidin antimicrobial peptide; FasL, Fas-ligand; IL, Interleukin; NETs, neutrophil extracellular traps; TGF, transforming growth factor; Th, T helper; TNF, Tumor Necrosis Factor; Treg, T regulatory.

Among the pleotropic effects of vitamin D, in the last few decades an increasing number of evidences suggested an intriguing link between vitamin D homeostasis and immune responses [17,18,19]. As such, many researchers speculated that autoimmune disorders, including type I diabetes, autoimmune thyroiditis, inflammatory bowel disease, rheumatoid arthritis, systemic lupus erythematosus, and multiple sclerosis, could be related to vitamin D imbalance [20,21]. In this context, vitamin D could exert an important role in innate and adaptive immunity modulation (Figure 1).

Innate immunity is an immunological subsystem that includes the cells and mechanisms implicated in the first line of defense from infections. The vitamin D binding to VDR expressed by the hematopoietic system leads to the myeloid differentiation towards monocytes and granulocytes, the immune cells involved in the innate immunity. The exposure of monocytes to different pathogens increases the expression of VDR, which is involved in antimicrobial response [22]. Focusing on innate immunity, 1,25(OH)2D3 enhances antimicrobial activity of monocytes and macrophages by promoting the production of defensin *β* 2 and cathelicidin antimicrobial peptide (CAMP) [23,24]. Furthermore, 1,25(OH)2D3 contributes to the clearance of pathogens by inducing chemotaxis and phagocytosis of innate immune cell components [25,26]. Recent evidences suggest that vitamin D seems to be implicated in the prevention of infections by reducing the propagation of pathogens, via neutrophil extracellular traps (NETs) formation [27]. Although vitamin D enhances the antimicrobial activity of innate immunity, it seems to exert an important role in favoring immune tolerance through the downregulation of antigen presentation by monocytes [28,29]. In addition, 1,25(OH)2D3 inhibits dendritic cells chemotaxis and antigen presentation, through a downregulation of MHC II expression [30,31].

Therefore, many studies highlighted an intriguing role for vitamin D in enhancing innate immunity through different pathways.

Adaptive immunity is highly specific for each pathogenic antigen and is mediated by lymphocytes B and T. With regards to the immunomodulatory effects of vitamin D on this subsystem, vitamin D downregulates the monocytes expression of proinflammatory cytokines, including Tumor Necrosis Factor *α* (TNF *α*) and Interleukin 6 (IL-6), which are involved in the inflammatory pathway that leads to B and T lymphocytes activation and proliferation [32]. B cells express VDR both in quiescence and after activation [33]. In this context, 1,25(OH)2D3 promotes the apoptosis B cells, hence preventing their proliferation and differentiation into plasma cells [34].

T lymphocytes represent another immune target of vitamin D action: 1,25(OH)2D3 is able to suppress T cells cytotoxic activity by inhibiting the expression of Fas-ligand and exert different immunomodulatory effects on T helper (Th) cells [35]. CD4+ T cells differentiate into several distinct subsets [36]. The Th1 subset secretes proinflammatory cytokines, including IFN-*γ* and IL-2, and exerts a key role in the clearance process of intracellular pathogens, whereas Th2 cells are involved prevalently in immune responses to parasites. Th17 cells secrete proinflammatory cytokines, such as IL-17 and IL-22, implicated in the immune responses to bacterial and fungal infections as well as in the pathogenesis of autoimmune diseases [37,38].

In animal models, 1,25(OH)2D3 regulates CD4+ Th differentiation, inhibiting the activity of Th17 and Th1 cells [39], which are involved in different chronic inflammatory conditions through cytokines release. On the contrary, 1,25(OH)2D3 polarizes CD4+ cells towards a Th2 phenotype with a consequent upregulation of cytokines including IL-4 and IL-5 [40,41]. Finally, 1,25(OH)2D3 has been shown to induce the cellular differentiation and increase the activity of T regulatory (Treg), a key subset of CD4+ cells implicated in the maintenance of immune tolerance. These mechanisms lead to an increase of anti-inflammatory actions mediated by transforming growth factor *β*1 (TGF-*β*1) and IL-10 and [42,43,44].

In summary, vitamin D has the ability to modulate adaptive immunity, acting on different components of this immunological subsystem.

The global biological actions of 1,25(OH)2D3 reveal, therefore, an ability to interact functionally with the immune system by promoting immune tolerance and a shift from the pro-inflammatory setting to a more tolerogenic immune setting, which may link to protective effects in autoimmune diseases and inflammatory processes [1,2]. Clinical surveys have recently associated hypovitaminosis D with autoimmune thyroid disorders (AITD), including Hashimoto’s thyroiditis (HT), Graves’ disease (GD) and post-partum thyroiditis (PPT), as well as thyroid cancer tumorigenesis [45,46].

This review aims to summarize the evidences on the immunomodulatory effect of vitamin D on thyroid diseases.

## 2. Autoimmune Thyroid Disorders (AITDs)

AITD is the most frequent autoimmune disease with an estimated prevalence of 5% and a progressive increase in incidence, especially in the female population. Adult women have a higher risk of developing thyroid autoimmunity than men and present more frequently abnormal thyroid function in this context (7%–9% in females vs. 1%–2% in males) [47]. AITDs are T-cell mediated autoimmune disorders, resulting from an organ-specific deregulation of the immune system. The mechanisms involved in this autoimmune response have not been fully elucidated yet, though an interaction between genetic predisposition and environmental factors has been demonstrated to trigger the autoimmune process [46]. In subjects with genetic predisposition, an alteration of the physiological balance between Th1 and Th2 response may occur in case of exposure to environmental factors [48]. Moreover, a shift in the balance between Th17 and Treg cells has been recently observed in thyroid autoimmunity [49]. Environmental factors that have been recognized in association with AITD pathogenesis include iodine, radiation, smoking habit, viral infections, drugs, and stress [50].

**Figure 2:**
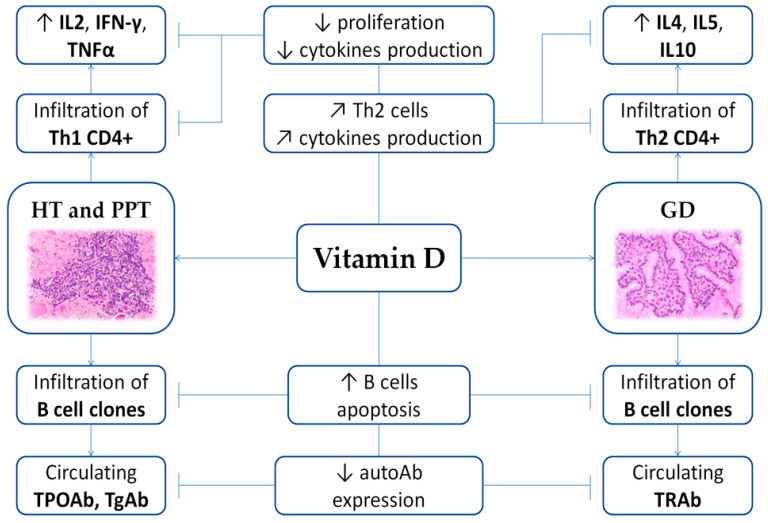
Scheme of the immunomodulating role of vitamin D on AITD. Arrows illustrate increase (↑), decrease (↓) or regulation/modulation (↗) of specific actions, processes, cells, or molecules. Abbreviations: autoAb, autoantibodies; GD, Graves’ disease; HT, Hashimoto’s thyroiditis; IFN, Interferon; IL, Interleukin; PPT, Post-partum thyroiditis; Th, T helper; TNF, Tumor Necrosis Factor; TPOAb, anti-thyroid peroxidase antibodies; TgAb, anti-thyroglobulin antibodies; TRAb, TSH receptor autoantibodies. Histological images are available at Histology Gallery, Yale Medical Cell Biology.

The most common AITDs are HT and GD, which are commonly characterized by lymphocytic (T-cell CD4+ and CD8+) infiltration of the thyroid tissue and production of thyroid-specific antibodies [48,51] (Figure 2). Patients with AITD harbor an increase of activated T-cell expressing human leukocyte antigen (HLA)-DR and a decrease of CD8+ immune cells, whereas circulating B cell levels are normal [51].

The HLA-DR antigen, expressed primarily by monocytes and B cells, has also been detected on the surface of activated T cells. These DR antigens, which are cell-surface glycoproteins encoded by genes of the HLA-DR region of the MHC, are absent in resting T lymphocytes and could represent a potential marker of the immune system activation [52]. Some studies also documented that the percentage of circulating T cells expressing HLA-DR represent a biomarker capable of accurately reflecting autoimmune diseases activity [53].

As previously described, vitamin D exerts a modulating role on AITD through its specific enhancing effects on the innate immune system and inhibitory actions on the adaptive immune response [2].

Preclinical and clinical studies found an association between AITD and vitamin D deficiency [45,54]. Original evidence of a peculiar role of vitamin D in thyroid disease dates back to the late 80s to early 90s. McDonnell described an interesting homology between the VDR and the thyroid hormone receptor [55], and five years later, Berg et al. demonstrated the VDR expression on follicular thyroid cells [56]. Moreover, VDR and the thyroid hormone receptor share partners for heterodimerization [57]. In the same period, Fournier et al. investigated the effect of a combined treatment with cyclosporine A and 1,25(OH)2D3 using an experimental model of AITD in mice [58], suggesting a synergistic effect of these molecules in preventing the onset of thyroid autoimmunity and its associated histological alterations [58]. Years later, Borgogni and colleagues evaluated the effects of a non-hypercalcemic vitamin D receptor agonist, elocalcitol, on the secretion of the inflammatory chemokine CXCL10 induced by proinflammatory cytokines, as compared to methimazole. The authors demonstrated that, in human thyrocytes, elocalcitol impaired both IFN-*γ* and TNF*α*-induced CXCL10 protein intracellular pathways, whereas methimazole only aced on IFN-*γ* pathway. Moreover, elocalcitol reduced Th1 and Th17 cytokine secretion in CD4+ T cells and promoted a shift toward a Th2 response [59].

In murine models with induced autoimmune hyperthyroidism prompted by thyrotropin receptor immunization, hypovitaminosis D was found to induce a persistent disease, suggesting an immunomodulatory effect of vitamin D status on autoimmune hyperthyroidism [60]. In parallel, Liu and co-workers tested the effect of 1,25(OH)2D3 on Th1/Th2 cells and inflammation in female Wistar rats with experimental autoimmune thyroiditis [61]. Their results showed significantly decreased levels of thyroid autoantibodies and INF-*γ* in mice treated with 1,25(OH)2D3, which was associated with the maintenance of structural thyroid integrity.

From a clinical viewpoint, a meta-analysis including 20 case-control studies showed that patients with AITD harbor significantly lower serum vitamin D levels compared to healthy controls (OR 2.99, 95%CI 1.88–4.74) [62]. However, the mechanisms underlying the effects of vitamin D on AITD are still unknown but likely related to its anti-inflammatory and immunomodulatory properties.

### 2.1. Hashimoto’s Thyroiditis

HT represents a T-cell-mediated autoimmune disease characterized by goiter, presence of circulating anti-thyroid peroxidase (TPOAb) and/or anti-thyroglobulin (TgAb) antibodies, and intrathyroidal infiltration of B and T cells with a CD4+ Th1 predominance [46,63]. This alteration leads to varying degrees of thyroid hypofunction.

Observational and interventional studies observed that low vitamin D levels and the risk of HT onset seem to be closely associated. Indeed, patients with HT harbored a high proportion of hypovitaminosis D (over 60%). Moreover, HT is more closely related to vitamin D deficiency (<20 ng/mL) than insufficiency (21–29 ng/mL) [64,65,66,67].

The first observational study on the association between vitamin D and HT was published in 2009 [68]. Based on the evidence that vitamin D deficiency is linked to a susceptibility to type 1 diabetes [69] and multiple sclerosis [70], Goswami et al. conducted a community-based survey on 642 adults to investigate the relationship between serum vitamin D concentrations and thyroid autoimmunity. Their results highlighted a significant inverse association between 25(OH)D3 and TPOAb levels [68]. Three years later, Camurdan et al. observed that hypovitaminosis D rate was higher in children with HT compared to control group (73.1% vs. 17.6%) and confirmed the inverse association between 25(OH)D3 levels and TPOAb titer in their pediatric population [71]. This inverse correlation was substantiated in the following studies: [66,72,73,74,75]. Furthermore, different clinical studies showed that the prevalence of HT in patients with hypovitaminosis D was significantly higher than that documented in subjects with sufficient vitamin D levels, particularly among children, elderly subjects, and pre-menopausal women [64,71,76,77,78,79,80,81]. As regards thyroid function in the context of HT, Mackawy and co-workers demonstrated a strong negative association between serum vitamin D concentrations and TSH levels, leading to speculate that vitamin D deficiency in HT patients could be associated with a progression towards hypothyroidism (TSH > 5.0 m UI/L) [65].

In more recent years, these evidences prompted several research groups to evaluate the effect of vitamin D supplementation on thyroid autoimmunity. Simsek et al. prospectively evaluated 82 patients with HT, which were randomized in two groups: the first group (46 patients) was treated with cholecalciferol 1000 IU/day for one month and the second group without vitamin D replacement. Their results showed that TPOAb and TgAb levels were significantly decreased by the vitamin D replacement therapy in the first group [82]. These findings were confirmed by other prospective studies and randomized controlled trials, which added evidence that cholecalciferol supplementary treatment was related to a decrease in TPOAb and TgAb levels both in patients with vitamin D sufficiency and deficiency [83,84,85]. Moreover, an increase of 5 ng/mL in vitamin D levels was correlated to a significant decrease of 20% in the risk of HT [86].

In 2017, Mirhosseini et al. enrolled 11,017 subjects to evaluate the influence of vitamin D supplementary treatment on thyroid function and thyroid auto-antibodies levels. Their results showed that serum 25(OH)D3 levels ≥ 50 ng/mL were associated with a 30% decreased risk of hypothyroidism onset and a 32% decreased risk of increased thyroid auto-antibodies levels, leading the authors to speculate that vitamin D supplementation could exert a positive effect on thyroid function as well as provide protection from new onset of thyroid disease during a 12 months follow up [87]. In addition, in a recent 3 month randomized controlled trial (RCT) on adult females with HT, Chahardoli et al. confirmed a significant decrease of TSH levels after weekly supplementation with 50,000 IU of cholecalciferol [88].

Few studies, however, failed to document associations between vitamin D deficiency and a higher prevalence of HT [89,90], questioning on the preventive role of vitamin D in AITD. Further investigations are needed to evaluate the preventive and therapeutic effects of vitamin D in HT.

Growing evidence also documented that some VDR polymorphisms could be related to an increased incidence of HT [91]. The most frequent polymorphisms include FokI, BsmI, ApaI and TaqI. FokI polymorphism is located in exon 2 of the VDR gene and causes an alteration in the start codon leading to a truncated VDR protein [92]. The BsmI and ApaI polymorphisms, located in intron 8 of the VDR gene, lead to an altered mRNA stability, a disruption of splicing sites or a change in intronic sequences, affecting gene expression [92,93]. The TaqI polymorphism is located in exon 9 and is able to alter the mRNA stability [92,93]. FokI and ApaI polymorphisms influences serum vitamin D concentration, and BsmI polymorphism interferes with the IFN-γ production by monocytes, whereas TaqI influences the VDR expression [92,93].

In a meta-analysis on 8 studies showed that the VDR BsmI and TaqI polymorphisms were associated with HT risk [94]. Later, Inoue and co-workers demonstrated that the CC genotype for the FokI polymorphism was frequent in patients with HT [93]. Finally, a meta-analysis including 11 studies on Asian and Caucasian populations observed that the FokI polymorphism of VDR was related with a higher risk of HT only in Asian subjects [95]. All these results are in line with findings on children with type 1 diabetes [96].

### 2.2. Graves’ Disease

GD is the most common cause of hyperthyroidism in developed countries, affecting mostly women, with an annual incidence of 14 cases in 100,000 persons [97]. GD is characterized by the presence of TSH receptor autoantibodies (TRAb) which lead to hyperthyroidism, diffuse toxic goiter, and ophthalmopathy [98]. In GD, infiltration of lymphocytes is milder than in HT and involves mainly CD4+ Th2 cells [46]. Although several studies reported an increased prevalence of hypovitaminosis D in patients with GD, the relationship between these two conditions is not clear [99].

The first observational study evaluated vitamin D status in women with and without GD remission. The results showed that vitamin D concentrations were significantly lower in patients without remission of GD compared to subjects with remission and that the prevalence of hypovitaminosis D was twice as high as in healthy controls [100]. The same workgroup, in a prospective study, observed a significant association between low vitamin D concentrations and an increased volume of thyroid gland in women with newly onset GD [101]. In 2016, Kim et al., in a cross-sectional study including 776 AITD patients, showed that the prevalence of vitamin D insufficiency was higher in GD patients compared to healthy subjects [79]. These results were further confirmed by two cross-sectional studies, although no association was observed between vitamin D and TRAb levels [102,103]. Conversely, in a cohort of 70 GD subjects, Zhang et al. found an inverse association between serum vitamin D concentrations and TRAb levels [104].

More extensively, Xu and co-workers evaluated the relationship between serum vitamin D levels and GD through a meta-analysis including 26 case-control or cohort studies. Their results confirmed that subjects with GD were more frequently to be deficient in vitamin D than the control group (OR = 2.24, 95% CI 1.31–3.81, *p* < 0.001) [105].

As regards the role of vitamin D supplementary treatment during GD, current evidence is limited to only one interventional study where the effect of daily vitamin D treatment was assessed on GD recurrence. Among 210 GD patients with hypovitaminosis D, 60 received cholecalciferol (1000–2000 IU per day) whereas 150 did not. Recurrence rate was comparable between groups (38% vs. 49%) but occurred earlier in the control group (7 vs. 5 months) [106].

Several studies investigated the relationship between polymorphisms of VDR gene and GD onset risk, but results remain arguable. The first meta-analysis to evaluate this association was conducted by Zhou et al. in 2009 and included seven studies on Caucasian and Asian populations. The results showed that the presence of ApaI, BsmI, and FokI VDR polymorphisms was associated with a higher risk of GD onset in Asian population, whereas no associations were found in Caucasian cohorts [107]. More recently, a meta-analysis including eight studies found a relationship between BsmI and TaqI polymorphisms and the risk of GD onset, while no correlation was seen for ApaI and FokI [94]. Finally, Inoue et al. observed a higher prevalence of TT genotype for TaqI in subjects with GD compared to patients with HT and a higher prevalence of the C allele for ApaI in comparison with controls [93].

### 2.3. Post-Partum Thyroiditis

Post-partum thyroiditis (PPT) refers to the development of de novo AITD within the first year post-partum and represents one of the most common autoimmune disorders in pregnancy, with an estimated prevalence between 1% and 17% [108]. Clinical symptoms include a thyrotoxic phase during the first 3 months of onset usually followed by a phase of hypothyroidism at 3–6 months, which is reversible in 75% of patients [109,110].

Different clinical studies investigated the relationship between PPT and serum vitamin D concentrations. Krysiak et al. compared 25(OH)D3 and PTH levels between 4 groups of non-lactating women who gave birth 12 months before the beginning of the study: euthyroid women with PPT, women with hypothyroidism and PPT, women with non-autoimmune hypothyroidism, and healthy euthyroid women without AITD. Serum vitamin D concentrations were lower whereas PTH levels were higher in patients with PPT compared to subjects without AITD. Moreover, in the second part of the study, women with hypothyroidism and PPT as well as women with non-autoimmune hypothyroidism were treated for 6 months with L-thyroxine. The results showed that L-thyroxine therapy increased serum vitamin D levels and reduced PTH levels only in the first group, highlighting an intriguing relationship between vitamin D status, PPT and L-thyroxine therapy [111].

In 2016, the same group investigated whether vitamin D treatment could modify the course of thyroid autoimmunity in 38 non-lactating levo-thyroxine-treated women with PPT compared to 21 matched healthy postpartum women. Women with deficiency of vitamin D were treated with oral cholecalciferol at 4000 IU daily, whereas women with insufficiency of vitamin D and women with normal 25(OH)D3 concentrations were either treated with cholecalciferol at 2,000 IU daily or left untreated. At baseline, serum vitamin D concentrations were lower in patients with PPT compared to healthy women and were inversely associated with thyroid antibody levels. Following vitamin D treatment, TPOAb titer decreased, and this effect was more evident in women with hypovitaminosis D compared to those with normal vitamin D [112]. However, this study raised some criticism regarding the presence of potential confounders that could interfere with autoantibody titer and the vitamin D status, including the use of estrogen contraceptives, iodine status, and selenium levels [113]. Further studies are needed to define the role of vitamin D in PPT.

## 3. Thyroid Cancer

Thyroid cancer is the most frequent endocrine tumor with 567,000 new cases reported annually. Its incidence is significantly higher in women than in men (10.2 per 100,000 vs. 3.1 per 100,000) [114]. Thyroid cancers are usually follicular in their origin, including differentiated thyroid cancers (DTC), poorly differentiated thyroid cancers (PDTC), and anaplastic (ATC) thyroid cancers [115].

Previous irradiation to the neck, the presence of benign thyroid nodules, and a family history of thyroid neoplasia represent recognized risk factors for thyroid cancer. Recently, a higher cancer risk for hypothyroidism and hyperthyroidism has been established [116,117,118,119,120]. An important role in thyroid tumorigenesis was also attributed to environmental factors, which can influence thyroid cancer histopathological phenotype [121,122]. In this context, obesity represents a recently recognized environmental and genetic risk factor involved in thyroid carcinogenesis. Several evidences suggest a potential role for adipose tissue in regulating tumor microenvironmental pathophysiology, supported by a documented association between obesity-dependent inflammation and cancer [123]. In fact, hypoxia, chronic inflammation, and oxidative stress, could favor the development of a subgroup of DTCs characterized by resistance to both 131I treatment and chemotherapy [124]. In the context of inflammation, some evidences indicate that HT is associated with a higher risk of PTC onset [125,126], resulting from an increased cytokines production which characterizes the autoimmune process [127].

**Figure 3:**
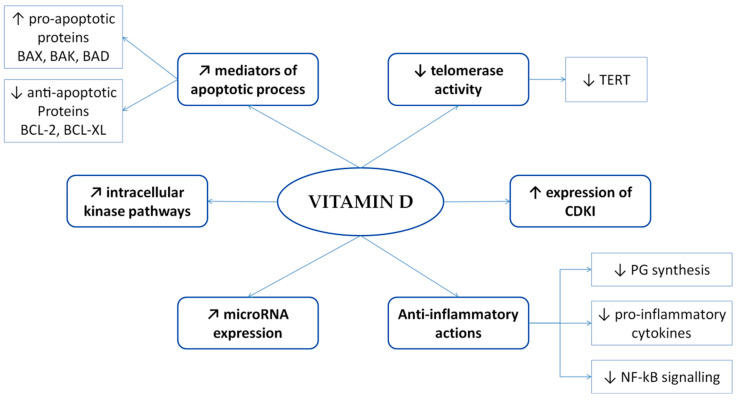
Scheme of the anti-neoplastic and anti-inflammatory role of vitamin D in thyroid tumorigenesis. Arrows illustrates increase (↑), decrease (↓), or regulation/modulation (↗) of specific actions, processes, cells, or molecules. Abbreviations: CDKI, cyclin dependent kinase inhibitors; PG, prostaglandin; TERT, telomerase reverse transcriptase.

The role of inflammation in DTCs has been focused on in several studies published in the last 10 years, demonstrating an intriguing relationship between chronic inflammation and increased risk of DTC and suggesting the role of inflammatory setting in cell transformation and tumor progression [128,129,130,131,132]. In this scenario, vitamin D seems to play a peculiar role in thyroid tumorigenesis for its immunomodulatory and antineoplastic properties. In fact, vitamin D can modulate many signaling pathways in apoptotic process, cellular proliferation and differentiation, angiogenesis, invasion, and inflammatory response [46,133] (Figure 3). In vitro and in vivo studies observed that vitamin D has pro-apoptotic, pro-differentiative, anti-proliferative and anti-inflammatory properties in the context of the tumor microenvironment [46].

More in detail, vitamin D regulates mediators of apoptotic process through activation of pro-apoptotic proteins (BAX, BAK and BAD) and inhibition of anti-apoptotic elements, such as BCL-2 and BCL-XL [134,135]. Moreover, 1,25(OH)2D3 increases cyclin-dependent kinase inhibitors (CDKI) expression and influences microRNA expression, which have a negative impact on cell proliferation [136,137]. In addition, 1,25(OH)2D3 modulates intracellular kinase pathways and inhibits the elevated telomerase activity of cancer cells by decreasing telomerase reverse transcriptase (TERT) [136,138].

Recently, several studies focused on the immunomodulatory role of vitamin D in tumor-associated inflammation. Vitamin D exerts beneficial anti-inflammatory properties in different cancer types through the inhibition of prostaglandin synthesis and signaling, the suppression of p38 stress kinase signaling with a consequent inhibition of pro-inflammatory cytokines production and NF-kB signaling [136,138]. As previously described, 1,25(OH)2D3 inhibits the proliferation and differentiation of Th1 and Th17 as well as the expression of IL-2, interferon-*γ*, IL-17, and IL-21, and promotes the expression of IL-3, IL-4, IL-5, and IL-10 [39,40,41,139]. On this basis, Passler and co-workers suggested that the inflammatory microenvironment in DTC could be reduced by 1,25(OH)2D3 [140].

While in clinical studies, hypovitaminosis D was associated with several types of cancers [141,142,143,144], controversial data are available about low vitamin D levels and thyroid cancer [137,145,146].

Basic studies seem to validate a role for vitamin D in thyroid tumor onset and progression. Anti-neoplastic actions are mediated by the binding of vitamin D to its receptor [145] and by interacting with other transcriptional factors or cell signaling pathways [147,148,149]. Available data on this topic suggest that local vitamin D could act in early cancer stage reducing proliferation and aggressiveness of thyroid tumors through different pathways. Khadzkou et al. observed an increased VDR and 1-alpha-hydroxylase expression in PTC specimens compared to the adjacent non-neoplastic thyroid tissue, particularly in areas with lymphocyte infiltration [145]. Likewise, an enhanced expression of the VDR and the two enzymes involved in vitamin D activation and degradation (CYP24A1 and CYP27B1, respectively) in surgical samples of follicular adenomas and DTC has been demonstrated, although a decreased expression of these genes was found in lymph nodal and distant metastases [150]. Moreover, expression of VDR was found to be reduced in lymph nodes metastases of PTC compared to normal thyroid tissue and primary PTC, suggesting that VDR expression and CYP27B1 could be predictors of a favorable prognosis [145]. In lymph node metastatic PTC, the expression of VDR and CYP24A1 was decreased compared to non-metastasized PTC, and the expression of VDR was frequently lost in ATC [146]. These observations were confirmed by Yavropoulou et al., who demonstrated an enhanced expression of both VDR and CYP24A1 in PTC samples than the adjacent non-neoplastic tissue [150]. Moreover, mRNA analysis allowed to demonstrate an increased expression of VDR in PTC, which is often linked to an increased expression of the type II trans membrane serine protease-4 and extracellular matrix protein-1, which are known to be important predictors of malignant thyroid nodules [151]. More recently, Zhang and co-workers observed a higher expression of VDR in PTC compared to adjacent non-tumoral tissue in group of 78 patients who underwent surgery. In the same cohort, pre-surgical serum concentration of 1,25(OH)2D3 was found to be lower in patients with PTC compared to patients with benign thyroid nodules [152]. Moreover, through a cyclic adenosine monophosphate-mediated process, 1,25(OH)2D3 inhibited the proliferation and induced the apoptosis of PTC cells [152]. On this path, numerous in vitro studies observed that the administration of 1,25(OH)2D3 is able to decrease proliferative activity of differentiated and undifferentiated thyroid cancer cells through different signaling pathways [153,154,155,156]. Liu and coworkers demonstrated that in vitro 1,25(OH)2D3 administration is able to increase the expression of p27 and to decrease cell proliferation in cultured thyroid cancer cell lines [157]. Subsequently, the same authors evaluated the in vivo effects of 1,25(OH)2D3 supplementation on thyroid cancer growth and progression in a xenograft model [158], demonstrating the restoration of p27 in thyroid cancer cells, an effect correlated to an improved cell differentiation and a preventive role on metastatic growth. Finally, animal studies showed that 1,25(OH)2D3 supplementary treatment was associated to a reduction of tumor volume [147].

These experimental results demonstrate that vitamin D status could exert an important impact on thyroid cancer progression and that 1,25(OH)2D3 could have a beneficial effect in thyroid cancer treatment.

Despite the evidence for anti-neoplastic effects of 1,25(OH)2D3 observed in vitro studies and animal models, clinical studies showed controversial results. Several studies found that lower 25(OH)D3 levels were significantly correlated to a higher risk of thyroid cancer onset [159,160,161,162,163,164] whereas others reported opposite results [165,166,167,168].

Most studies observed significantly lower serum 25(OH)D3 concentrations in patients with DTC than individuals with benign thyroid diseases or healthy controls [159,160,162,163,164]. A recent meta-analysis including 14 case-control studies showed that pre-surgical serum 25(OH)D3 levels were lower in patients with thyroid cancer than controls, but this difference disappeared after surgery [133]. Similar results were reported by Hu et al. in meta-analysis that included 10 case-control studies, demonstrating a higher risk of thyroid cancer in individuals with hypovitaminosis D [169]. A negative prognostic role of vitamin D has also been supposed, since low 25OH-D3 levels were found to be associated with advanced disease and aggressive clinical-pathologic features [164,170,171].

Another point of discussion is the finding of a reduced conversion of 25(OH)D3 to 1,25(OH)2D3 in DTC patients, that leads to speculate a potential role of CYP24A1 gene polymorphism in thyroid carcinogenesis [161]. In fact, in recent years, Zhang et al. demonstrated lower 1,25(OH)2D3 levels in PTC compared to nodular goiter [152].

Finally, a few clinical studies evaluated the role of vitamin D supplementation in preventing thyroid cancer onset. In 2013, a systematic review on 11 studies was conducted to evaluate the relationship between dietary supplements of vitamins and minerals, including vitamin D, and the risk of thyroid cancer [172]. The results suggested that the current evidences supporting a protective role of vitamin D on thyroid cancer onset are inconclusive. One year later, the prospective US National Institutes of Health American Association of Retired Persons (NIH-AARP) Diet and Health Study did not show any clear evidence of positive or negative correlation between dietary intake of vitamin D and thyroid cancer risk [173]. No human studies on 25(OH)D3 and 1,25(OH)2D3 supplementations have been conducted yet.

Lastly, there is an underlying possibility that discrepancies existing among different studies on vitamin D role in thyroid function, autoimmunity, and cancer could depend on inter-laboratory and inter-assay variability in the methods used to measure 25(OH)D3, as well as seasonal variations of serum 25(OH)D3 concentrations and differences in the 25(OH)D3 reference levels used to define hypovitaminosis D. Moreover, the controversial results could be attributed to the cross-sectional design of studies with a low sample size and a heterogeneous population [46].

## 4. Conclusions

In conclusion, several studies observed a relationship between hypovitaminosis D and thyroid diseases. Supplementary treatment with cholecalciferol seems to have beneficial effects on AITD, whereas there are no clear evidences on a correlation between vitamin D supplementation and thyroid cancer risk. However, large multicenter studies are needed to investigate the impact of vitamin D supplementary treatment on meaningful long-term clinical end points in AITD and thyroid cancer.

## Abbreviations

**Abbreviation**	**Definition**
1,25(OH)2D3	1,25-dihydroxyvitamin D
25(OH)D3	25-hydroxyvitamin D
AITD	Autoimmune thyroid disorders
ATC	Anaplastic thyroid carcinoma
AutoAb	Autoantibodies
CAMP	Cathelicidin antimicrobial peptide
CDKI	Cyclin dependent kinase inhibitors
DTC	Differentiated thyroid cancers
FasL	Fas-ligand
FGF23	Fibroblast growth factor 23
GD	Graves’ disease
HLA	Human leukocyte antigen
HT	Hashimoto’s thyroiditis
IFN	Interferon
IL	Interleukin
MHC	Major histocompatibility complex
NETs	Neutrophil extracellular traps
PDTC	Poorly differentiated
PG	Prostaglandin
PPT	Post-partum thyroiditis
PTC	Papillary thyroid cancer
PTH	Parathyroid hormone
RCT	Randomized controlled trial
TERT	Telomerase reverse transcriptase
Th	T helper
TGF	Transforming growth factor
UVB	Ultraviolet B
TgAb	Anti-thyroglobulin antibodies
TNF	Tumor Necrosis Factor
TPOAb	Anti-thyroid peroxidase antibodies
TRAb	TSH receptor autoantibodies
Treg	T regulatory
VDR	Vitamin D receptor
VDRE	Vitamin D response element
